# Case of Hysteritis

**Published:** 1840-03-14

**Authors:** Benjamin Fleet

**Affiliations:** King and Queen, Va.


### CASE OF HYSTERITIS.

BY BENJAMIN FLEET, M. D.

On Saturday, February 15th, I was called to visit Mrs. W——, aged about thirty-five, labouring under a most violent hysteritis. She had her fifth child eight days previously to my being called in, and was quite plethoric, the child larger than any she had ever before borne.

*Symptoms, as presented in her case.*

Found the patient labouring under the most violent pain over the whole of the lower part of abdomen, and it also extended itself to the lumbar region. Entire inability to move any part of her, but hands and arms, without an evident aggravation of the pain. Lochiæ rather suppressed, and not of natural colour, rather paler, perhaps, than naturally. Urine scanty and high coloured, of course voided with great difficulty. Bowels constipated. Pulse from ninety to one hundred beats to the minute, and rather tense than otherwise. Tongue loaded with a whitish viscid fur.

*Treatment.*—Venesection was practised to ℥xx. Pulse rather reduced in fulness, as also tenseness and frequency. Three hours afterwards examined my patient, and found that the pulse had reacted, quite as full as before venesection had been practised. Venesection again to ℥xiv. About 9 o'clock left, and prescribed gr. x pulv. Dov., with xii proto chloride of mercury. Perhaps she was a little more free from pain. Fomentations of flannels, wrung out of hot brandy, directed to uterine region. Took, in the morning, two tablespoonfuls of ol. ricini, with a teaspoonful of magnes. calc. opt.

*Sunday, 16th.*—Called at 10 o'clock, and found her a little easier, but still great pain over lower part of abdomen; she could move herself rather more, and not so much pain on taking a full inspiration. Medicine has not operated; pulse about the same as yesterday; venesection was again carried to ℥xvj. At half past 12, M., left. At 4 o'clock called again; found the blood that had been drawn in the morning, and the evening before, covered with a thick buffy coat, not less than from one-half to three-fourths of an inch in thickness, and that below exceedingly black. She had been taking barley and gum water freely during the day. The medicine she took this morning operated well. Pulse, this afternoon, continues full, and about the same number of beats as this morning. Venesection again to ℥viij, from the same orifice. At 6 o'clock left, and prescribed gr. x Dov. pulv., to be taken "pro re nat." Continue the use of the gum and barley water freely, and if her appetite improves allow her a little stale bread, crumbled in hot water tea.

*Monday morning*, 17*th*.—Found patient decidedly improved; slept so well as not to require the anodyne. She had quite a copious evacuation during the night. Pulse, this morning, one hundred, but not quite as tense and full as before. Venesection to ℥xiv, and with more decided effect on the pulse than any previous bleeding; blood improved much in appearance, but still inflammatory.

I should have mentioned that the *fomentations* yielded *no earthly relief*, that I could perceive, and I, of course, discontinued them; they may have contributed somewhat to have mitigated the symptoms. Two other facts which I have left out are, that fortunately there was no irritability of stomach in this case, nor had the disease extended itself to the peritoneum, although she had been labouring under it some three days before I saw her.

This morning she took pulv. rhei. ℥ss, in combination with ʒi mag. calc. opt., which had not operated when I left, (11 o'clock.)

Directed an enema of common gruel, in combination with sulph. mag., if the cathartic did not operate by 12 o'clock, M.

*Tuesday morning*, 18*th*.—Repeat the cathartic of rhubarb and magnesia, and give the anodyne of pulv. Dov. at night, "pro re nat."

*Wednesday morning*, 19*th*.—Called at 11 o'clock, and found patient considerably debilitated, though the soreness over the uterus had not entirely subsided. Venesection was again carried to ℥xiv. Tongue to-day is slightly coated with a white fur. ℞.—Proto-chlorid. merc. et comp. ext. colocynth, $\overline{aa}$ gr. x, ft. pil. iii, to be taken at intervals of an hour, about bedtime. Ol. ricini. in the morning, should the pills not operate well.

20*th*.—Called at 12 M., found patient's pulse one hundred to one hundred and eight; tongue improved; pain and soreness over uterus entirely subsided, but still some soreness over both iliac regions. Directed an epis. over both regions. The calomel and comp. ext. colocynth, brought away quite bilious discharges.

The last bleeding, I should have remarked, was decidedly less inflammatory than either of the preceding. Difficulty of evacuating contents of bladder entirely subsided.

22*d*.—Called again this morning and found patient decidedly worse than when I last saw her. She was permitted to partake of rich chicken soup in my absence, which excited her so violently as to render her unable to sleep during the greater part of last night. Partook of it again to-day, just before I arrived. Found her with a corded, tense and full pulse, number of beats to the minute, 104. Pain over uterus almost as violent as at first. Tenderness over iliac regions still continues, although the blisters have drawn well. Venesection was again carried to ℥xiv. Blood very inflammatory, thickly coated with buff, and somewhat cupped. Gave her cit. potass. in combination with ant. tartariz. and ol. limon, as a febrifuge, a table spoonful of the mixture to be taken every two hours. Ordered an enema of ol. ricini, in combination with flaxseed tea, to be followed by an opiate to-night, magnesia and rhubarb in the morning. Discontinue soups, live on barley and gum water, together with arrow root jelly.

23*d*.—Found patient this morning more cheerful and better; pulse one hundred, soft and compressible; pain and soreness not entirely subsided. Venesection to ℥viij was performed. Difficulty of passing contents of bladder still continues. Ordered an infusion of garden parsley, with water-melon seed, and the spir. ætheris nitros. added to it, to be taken freely, which had the effect of removing the difficulty. Take to-night proto-chlorid. merc. and pulv. opii, gr. xv of the former with two of the latter. Divide into two pills, one at 7 and the other at 9 o'clock in the morning. Bitart. potass. ℥i, and tart. potass. et sod. ℥iss.

25*th*.—Called this morning, and found patient *decidedly improving*. Pulse one of debility, more than of febrile action, number of beats one hundred; tongue moist and clear; pain and soreness nearly subsided. The medicines she took yesterday had the effect of producing a copious discharge of urine. Slept well last night, but was quite exhausted by the operation of the medicines she took yesterday. Continue low diet, take an enema of ol. ricini and flaxseed tea this afternoon, "pro re nat." Bowels to be kept soluble with bitart. potass. and tart. potass. et sod.

28*th*.—Called and found all the disagreeable symptoms had subsided, and patient evidently recovering. Ordered a wine glass full of the infusion of chamomile to be taken three times a day, and more nourishing diet.

The patient now (March 16th) is well, and and attending to her domestic matters.

*King and Queen, Va., March* 16th, 1840.

